# The International Medical Annual and Practitioners’ Index

**Published:** 1895-09

**Authors:** 


THE INTERNATIONAL MEDICAL ANNUAL AND PRACTITIONERS' INDEX. A work of reference for medical practitioners. Thirteenth year. Price, $2.75. New York: E. B. Treat, 5 Cooper Union. Chicago: 199 Clark street. 1895.

The annual for 1895 is in general appearance, style and make-up similar to its predecessors and needs no introduction to the medical public. The new features for 1895 are many and varied, making it superior to the former volumes. The therapeutic review of the past year and dictionary of new remedies comprise part first. The present status of electro-therapeutics is ably discussed by Dr. A. D. Rockwell, mention being made to the sinusoidal current of D'Arsonval's which possesses greater penetrating power than the Faradic and is attended with less pain. The chapter following on anti-microbic treatment by Prof. Alfred Carter is well written, but the subject is too specialised.

The bulk of the volume is taken up with a dictionary of new treatment in medicine and surgery, written by an able staff of contributors. Among the articles which deserve special praise is that on angio-neuroses, by Ramsay Smith ; methodical diagnosis of ear diseases, by J. Dundas Grant; eyesight as influenced by school life, by Simeon Snell, a most admirably written and illustrated paper on school room hygiene; Fredreick's disease, by Hector Mackenzie ; idiocy, by G. E. Shuttleworth, including a report of cases of cretinism, treated with thyroid extract; infantile paralysis, with numerous illustrative cases, by Robert Jones, of Liverpool, and John Ridlon, of Chicago; pyorrhea alveolaris, by J. Fitzgerald ; and other articles of less extended research and study. The closing chapters on new books, new instruments, hospitals, etc., are brought down to date and are exceedingly convenient. W. C. K.

